# Cancer Sample Biobanking at the Next Level: Combining Tissue With Living Cell Repositories to Promote Precision Medicine

**DOI:** 10.3389/fcell.2019.00246

**Published:** 2019-10-22

**Authors:** Hella A. Bolck, Chantal Pauli, Elisabeth Göbel, Katharina Mühlbauer, Susanne Dettwiler, Holger Moch, Peter Schraml

**Affiliations:** Department of Pathology and Molecular Pathology, University Hospital of Zürich, Zurich, Switzerland

**Keywords:** tissue biobanking, living cell biobanking, patient-derived tumor models, organoids, personalized medicine

## Abstract

Biorespositories of formalin-fixed and paraffin-embedded (FFPE) or fresh frozen human tissues from malignant diseases generated as integral part of the diagnostic workup in many pathology departments have been pivotal resources for translational cancer studies. However, such tissue biobanks have traditionally contained only non-viable specimens and thus cannot enable functional assays for the discovery and validation of therapeutic targets or the assessment of drug responses and resistance to treatment. To overcome these limitations, we have developed a next-generation comprehensive biobanking platform that includes the generation of patient-derived *in vitro* cell models from colorectal, pancreatic and kidney cancers among others. As such patient-derived cell (PDC) models retain important features of the original human tumors, they have emerged as relevant tools for more dynamic clinical and experimental analyses of cancer. Here, we describe details of the complex processes of acquisition and processing of patient-derived samples, propagation, annotation, characterization and distribution of resulting cell models and emphasize the requirements of quality assurance, organizational considerations and investment into resources. Taken together, we show how clinical tissue collections can be taken to the next level thus promising major new opportunities for understanding and treating cancer in the context of precision medicine.

## Introduction

Despite significant progress made in understanding and treating cancer in the last decades, this disease is still one of the leading causes of mortality worldwide (Siegel et al., 2016). Progress in cancer management has recently resulted from the advent of new sequencing technologies that have made it increasingly feasible to identify cancer-specific genomic alterations. Even though, this has provided rationales for the development of novel targeted therapies, the average number of new drugs approved for cancer therapy has surprisingly declined at the same time (Hay et al., 2014). A major reason for this apparent failure to translate promising drug candidates from the bench to the bedside can certainly be attributed to the inadequate use of model systems for human cancer research. To date, pre-clinical studies and screening programs of potential anti-cancer drugs have mostly utilized immortalized cell lines which often fail to recapitulate fundamental biological features of human tumors (Beroukhim et al., 2009; Horvath et al., 2016). Realizing these inherent limitations, there has been a growing need for more pathophysiological relevant cell culture tools that can be used to study tumor biology and consequently to rationally design precision treatment strategies.

Patient-derived cell (PDC) models generated from surgical biopsy specimens have recently emerged as desired pre-clinical tools. PDC models are characterized by increasing complexity and biological relevance as they retain patient-specific features and are amenable to a variety of experimental applications (Pauli et al., 2017; Drost and Clevers, 2018; Vlachogiannis et al., 2018; Bolck et al., 2019). Thus they have the potential to functionally complement molecular and pathological tumor analysis and advance precision oncology while at the same time fulfilling the current need for more relevant cell culture systems for cancer research.

In the Department of Pathology and Molecular Pathology at the University Hospital Zürich, we have developed a comprehensive biobanking platform that has evolved from our extensive routine sample collection of biospecimens from malignant human tumors and includes the generation of patient-derived *in vitro* cell models. Here, we describe the main aspects of this biobanking strategy detailing processes such as the acquisition of PDCs, and the generation, annotation, characterization and distribution of resulting cell models. We discuss in detail how clinical tissue biorepositories can be advanced to enable functional studies and personalized oncology and relate to our experience in developing a living cell biobank alongside the diagnostic workup.

## A Comprehensive Biobanking Strategy Offers Novel Opportunities for Basic, Translational and Clinical Research

**FIGURE 1:**
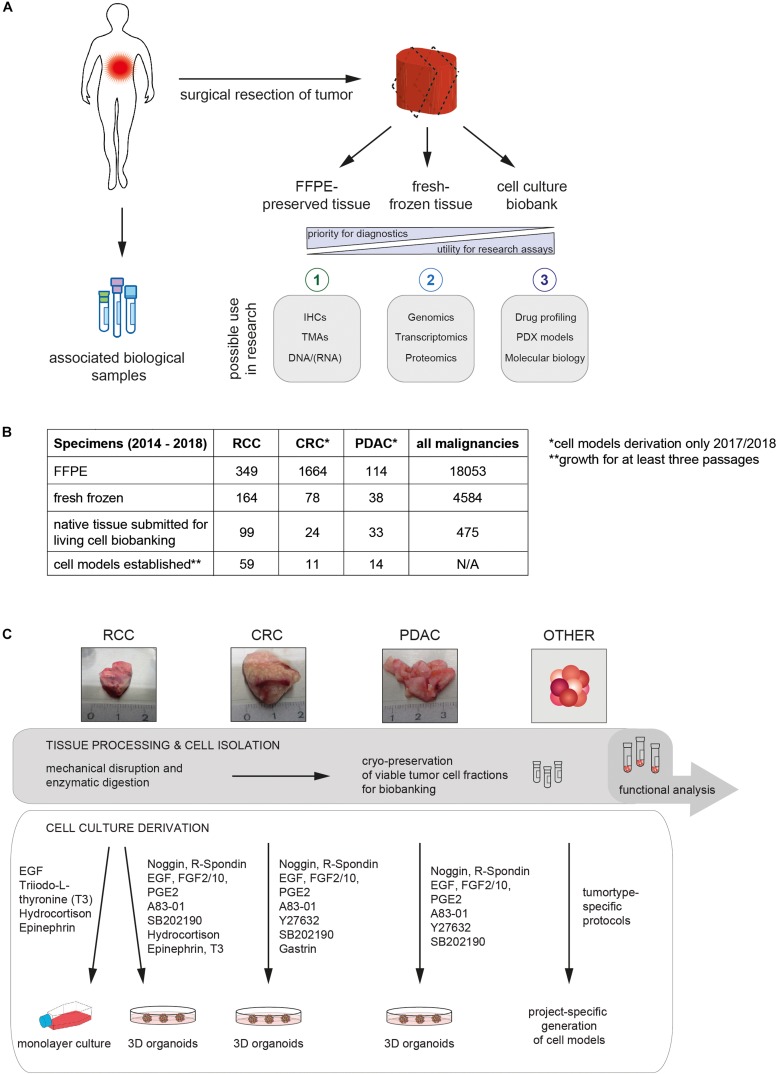
Collection of human tumor tissue for diagnostic purposes and biobanking. **(A)** Diagram summarizing the workflow for surgical specimen and biopsy processing in the Department of Pathology and Molecular Pathology (University Hospital Zürich). Fresh tissue is sectioned and consequently formalin-fixed and paraffin-embedded (1) or fresh frozen (2). If available, the remainder of tumor tissue is processed in the living cell biobank to establish 2D and 3D cell models (3). Fresh tumor tissue and cells from established culture models are viably frozen for biobanking purposes. In addition, the repositories of fixed and cryopreserved tumor-derived biospecimens can be further extended, e.g., by collecting matched body fluids (e.g., blood or urine). **(B)** Number of cases in which tumor samples were collected by the tissue and living cell biobanks of the Department of Pathology (University Hospital Zürich) from January 2014 to December 2018. Numbers for patients with specific diagnoses that were included in living cell biobanking are indicated separately (renal cell carcinoma [RCC], colorectal cancer [CRC] and pancreatic ductal adenocarcinoma [PDAC]). A summary of case numbers encompassing all malignancies which were considered for biobanking are presented as a reference. Native tissues collected for living cell biobanking from other malignancies than RCC, PDAC and CRCs were not always taken into culture. Instead viable cells were cryopreserved and thus the number of established cell models was not determined (N/A). **(C)** Diagram depicting the generation of PDC models from native patient tissue using tumor type-specific media components. Representative images of surgical specimens obtained for living cell biobanking are shown in the top panel.

Hundreds of clinical samples are obtained every day in routine pathology practice where they are processed for diagnostic purposes. Generally, a large proportion of these samples originates from surgical resections of tumors. These tissue specimens are routinely archived as FFPE blocks, the gold standard material for histological and immunohistochemistry analysis and simultaneously assembled into biobanks that are stored for many years (Moch et al., 1999; Schraml et al., 1999; Bubendorf et al., 2001). These pathology collections have been the most prevalent in the origins of biobanks as they have formed the basis for patient diagnoses. Out of the recognition that such collections of annotated samples can provide an excellent resource for biomedical research, tumor tissue biobanking has been constantly growing and gaining importance (Vaught, 2016; Coppola et al., 2019). With the advent of molecular pathology, snap freezing of OCT-embedded pieces of adjacent cancer and normal tissue have become critical aspects of tissue biobanking (Steu et al., 2008) because these specimens allow more comprehensive genomic, transcriptomic or proteomic studies (Figure 1A). As pathological examination of fresh frozen tissue samples is usually not part of the routine diagnostic workflow, Hematoxylin and eosin (HE)-stained cryostat sections need to be reviewed by a designated pathologist to ensure that morphological parameters of the frozen sample such as tumor cell content, stromal components, and presence of necrosis are recognized before embarking on sophisticated molecular profiling studies. Importantly, with these approaches, thousands of samples have been collected by the tissue biobank at our department. This is illustrated by the number of specimens that were stored in the five year period (beginning of 2014 to the end of 2018) that we focus on in this article (Figure 1B).

The retrospective analysis of these high-quality and readily available repositories that are linked with relevant clinical and pathological information allows the definition of cohorts for example for the creation of tissue microarrays or libraries of high quality nucleic acids for high-throughput molecular analysis (Figure 1A). Thus, these are extremely valuable for the evaluation of tumor subtypes, cellular and molecular heterogeneity and for the discovery and validation of biomarkers. However, given that all tissue is fixed or frozen and thus non-viable, such traditional tissue biobanks cannot provide tools for the discovery and validation of therapeutic targets or the assessment of drug response, cancer progression or resistance to treatment. In order to provide pre-clinical cancer models that are amenable to functional assays, we have complemented our large longitudinal collection of formalin-fixed tumor biopsies and fresh frozen tissues with viable PDC models (Figure 1A). In the subsequent sections, we describe main aspects of the extensions we made to traditional tissue collection with the aim to establish a living cell biobank of patient-derived *in vitro* cell models in order to promote cancer research and precision medicine.

## Technical, Organizational and Procedural Requirements for Establishing a Living Cell Biobank

Even more than traditional biobanking, the generation and maintenance of a living cell biobank requires a significant regulatory, administrative and laboratory infrastructure and the coordinated efforts of multiple teams. Setting defined criteria for the identification of tumor specimens that should be included into living cell biobanking was a first essential step to manage the workflow as it was neither feasible nor practical to collect samples from all tumor patients. In our living cell biobank, we specifically collect specimens from tumor types that will be integrated into running projects by our collaborating investigators. These have predominantly included renal cell carcinoma (RCC), pancreatic ductal adenocarcinoma (PDAC) and colorectal cancer (CRC). Other tumor types have been considered but still play a minor role for our biobanking activities and will not be further described here. Besides being the tumor type of our interest, specimens entering the living cell biobank should ideally have a minimum size of about 0.5 cm^3^. Even though establishing cell models from smaller specimens can be successful, this ensures that some of the original material can be viably frozen in parallel with cell culture derivation. In daily practice, the size of suitable tumor specimens usually ranges between 0.5 and 8 cm^3^ (Figure 1C). Upon first screening, we also consider that patients may have infectious diseases such as Hepatitis, HIV or tuberculosis. Processing samples from these patients can potentially pose a risk to research personnel and should therefore not be performed. Of note, a large proportion of specimens cannot be included in living cell biobanking because the resected tissue is required entirely for diagnostic purposes, which have priority over biobanking and research. Thus, only a subset of tumors could be subjected to PDC culture generation each year (Figures 1A,B).

**FIGURE 2:**
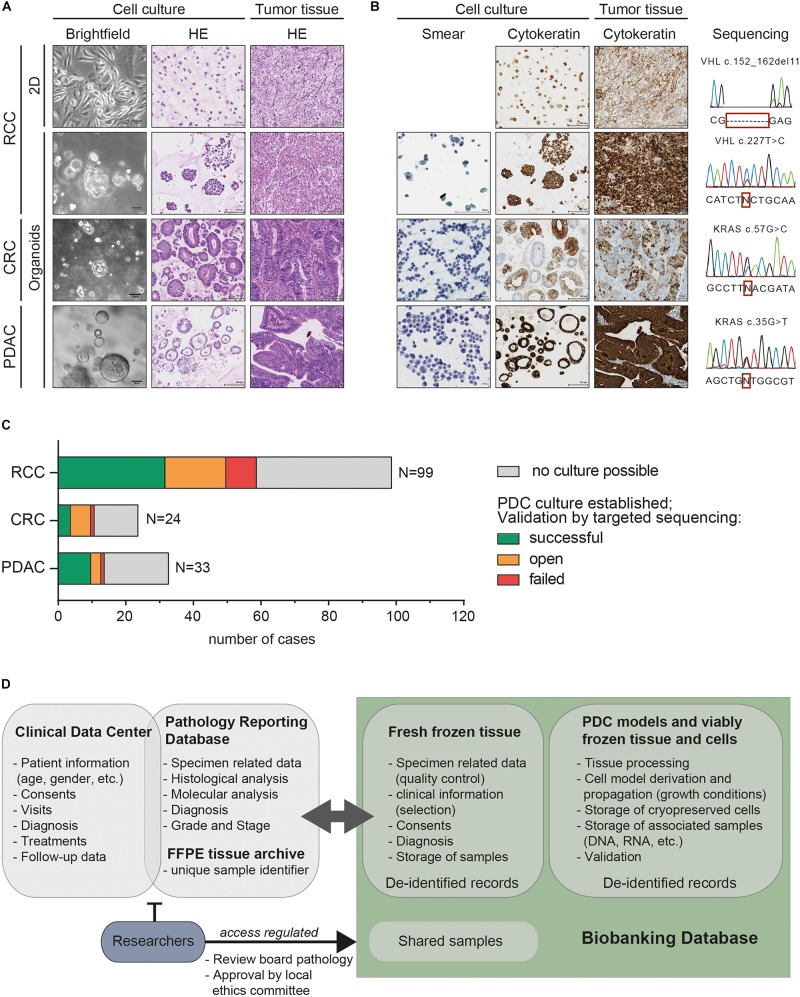
Comprehensively characterized patient-derived cancer models are collected for the living cell biobank that is accessible for cancer research and precision medicine. **(A)** Representative bright field microscopy of patient-derived models from renal cell carcinoma (RCC), colorectal cancer (CRC) and pancreatic ductal adenocarcinoma (PDAC) depict gross morphology during *in vitro* cell growth (X10 objective, scale bar denotes 50 μm). HE-stained slides show histology of the tumor monolayer or organoid cultures in comparison to matched tumor tissues demonstrating that histopathologic features are largely preserved (X10 objective, scale bar denotes 100 μm). **(B)** Pap–stained cytological smears (X20 objective, scale bar denotes 100 μm) of tumor organoids indicate characteristic features of malignancy. Immunohistochemistry (IHC) staining displaying concordant expression of cytokeratins (pan-Cytokeratin for RCC and CK19 for PDAC and CK20 for CRC specimens) in the cell models and matched patient tumor tissues (X10 objective, scale bar denotes 100 μm). Cancer driver mutations characteristic for each case were present in the original tumor and patient-derived cell models. Representative Sanger sequencing profiles are shown for each case. **(C)** Bar chart of surgical specimens that were considered for the generation of PDC models over 5 years. Cell model derivation was not possible if no viable cells remained following tissue processing or cells did not proliferate sufficiently for at least three population doublings (gray – no culture possible). For further validation, primary tumor samples and corresponding PDC models were subjected to targeted sequencing analysis of cancer driver genes. This included the assessment of *VHL* mutations for clear cell RCCs, *MET* amplifications in papillary RCCs and *KRAS* mutations in PDACs and CRCs. The number of PDC cultures retaining the alterations of the original human tumors is indicated (green - successful). Tumors and corresponding cell cultures without known cancer mutations were not comprehensively analyzed (orange - open). PDC models that did not retain the mutation of the human tumor are marked as “failed” (red). **(D)** Diagram depicting the general structure of the databases used for biobanking. Diagnostic and patient related data is available through the clinical data center and the pathology reporting database. These contain sensitive patient data and are therefore not accessible for external researchers. The designated biobanking IT infrastructure collects all patient and sample related information in a pseudonymised way. Researchers who have obtained ethical and review board approval can obtain FFPE and fresh frozen tissue as well as PDC models through this biobanking infrastructure.

If sufficient tissue was available, suitable pieces were selected for living cell biobanking by a board-certified pathologist and subsequently collected by the biobanking team in cold, sterile RPMI medium. Subsequently, all specimens were subjected to mechanical disaggregation and afterwards further processed according to previously described methods (Figure 1C; Boj et al., 2015; van de Wetering et al., 2015; Pauli et al., 2017; Bolck et al., 2019). Tumor organoids were generated from CRC and PDAC samples using a 3D cell culture system based on growth factor-reduced Matrigel, while RCC-derived cells were subjected to monolayer cell culturing and tumor organoid formation in parallel (Figures 1C, 2A). This strategy was based on our previous findings that both 2D and 3D PDC models capture important biological features of human clear cell RCC (Bolck et al., 2019). In this context, it is important to note that even though the information represented by 2D and 3D cell cultures can vary, both models offer important insights into tumor biology (Xu et al., 2014; Stock et al., 2016; Carragher et al., 2018; D’Agosto et al., 2019). Thus, while there has been no demand for 2D *in vitro* models of CRC or PDAC, we continue to rely on monolayer cell culture systems for models derived from human RCC tissue.

A total of 99 RCCs, 24 CRCs and 33 PDACs were subjected to PDC cultures derivation from 2014 to 2018. *In vitro* cell models could be established from 60% of the RCC, 46% of the CRC and 42% of the PDAC tissue samples (Figure 1B). According to our observations, lack of viable cells upon culture initiation or no significant cell proliferation after culture derivation were the main reasons for the initial failure to generate PDC models. In addition to establishing cell culture models, fractions of the dissociated cells or disaggregated clusters from primary tissues were routinely cryopreserved as a reference and for “future-proofing” in order to ensure that emerging technologies can be set up using the native tumor populations (Figure 1C).

In general, the protocols for PDC model derivation, propagation and collection of derivatives for characterization and other experiments (e.g., cell pellets, DNA extractions, FFPE embedded cell pellets, etc.) were subject to project-specific considerations that we undertook in collaboration with the principle investigators and which preceded any research project involving PDC models from our living cell biobank. In the last years, 3D tumor organoids have been usually maintained under serum-free conditions with growth factors that mimic the *in vivo* stem cell niche (e.g., the Wnt-agonist R-spondin 1, the transforming growth factor beta (TGF-b) inhibitor Noggin) (Figure 1C; Boj et al., 2015; van de Wetering et al., 2015; Baker et al., 2016). Cells derived from RCCs frequently grew in monolayers requiring a reduced set of growth factors (Figure 1C). These growth conditions stimulated proliferation for only shorter periods and PDC cultures usually became senescent after approximately 10 passage doublings. However, as early passages resembled the original tumor closely in terms of genetic and cellular heterogeneity, these can still provide a simple and robust model system for studying specific aspects of tumor biology (Bolck et al., 2019). Growth rates were strongly variable between patient-derived samples. Once established, PDC cultures were expanded by serial passaging for at least three population doublings with the goal of cryopreserving viable cells for biobanking purposes. Nevertheless, most cell models continuously proliferated for more than 5 passages and a significant fraction have been maintained in culture beyond passage 20.

## Stringent Characterization and Validation Ensures PDC Models are Representative of the Human Tumor

During the propagation phase, morphologies of PDC models were continuously assessed by bright-field microscopy and typical phenotypes like the glandular structures displayed by many CRC-derived organoids were recorded (Figure 2A). In addition, stringent validation and characterization was required before initiating additional analyses to ensure that the PDC models retained specific features of the original human tumors and were not contaminated with non-tumor or stromal cells. An initial histopathological analysis of HE-stained tissue and tumor model sections benefited from the key expertise in pathology in our department. It could often already provide first confirmation of the presence of typical cancerous features such as cells with enlarged and polymorphic nuclei or conspicuous nucleoli (Figure 2B). Cytological smears prepared from tumor organoids presented another quick method to screen whether these PDC models contained cells with malignant features (Figure 2B; Pauli et al., 2016). To further verify that PDC models showed concordance with the original tumor tissue, protein expression patterns were assessed by immunohistochemistry (IHC). To illustrate this method, expression of cytokeratins (CK19, CK20 or pan-cytokeratin) is shown in Figure 2B which confirms the epithelial origin of patient tumors and matched PDC models.

Even though we found IHC analysis to be a suitable tool to verify the origin of cell type, according to our observations it was often insufficient to unequivocally distinguish cancer cells from normal epithelial cells. The analysis of genetic cancer driver events was a more reliable method to exclude that PDC cultures contained predominantly benign epithelial cells. Extensive genomic profiling by whole-genome or whole-exome DNA sequencing of the original tumor and corresponding PDC models, preferably over several passages and after freeze-thaw would be the gold standard to confirm that cell models represent the mutant cancer cells. With such approaches, it is not only possible to unambiguously verify whether the genomic profiles of tumors and corresponding *in vitro* cell models match but additionally, details about subclonal composition and their dynamics can be deducted from the large-scale sequencing datasets. This potentially allows grouping cases according to mutational signatures or even unveils information about treatment targets (Sachs et al., 2018; Tiriac et al., 2018). However, extensive molecular sequencing is costly and requires advanced bioinformatics analysis. In our living cell biobanking workflow, we therefore routinely employ less sophisticated methods such as targeted sequencing for PDC model validation (Figure 2B). In cases for which characteristic molecular features, such as point mutations in tumor-associated genes were known from the clinical workup, this straight-forward approach was sufficiently informative and could confirm that PDC models retained the cancer driver events. Here, this is exemplified by the strategy we have employed for cell models derived from clear cell renal cell carcinoma (ccRCC). Importantly, ccRCCs often harbor mutations in the *VHL* tumor suppressor gene (80–90% of ccRCCs) that can be easily analyzed by targeted sequencing (Rechsteiner et al., 2011). In the 5-year period, in which we derived *in vitro* models from ccRCCs, we could confirm the presence of the *VHL* mutation of the parental tumor in 75% (28/37) of the corresponding PDC cultures by Sanger sequencing. Similarly, we assessed activating mutations in *KRAS* that are common in CRC (30–40% of cases) and almost ubiquitous in PDAC (Kandoth et al., 2013; Ying et al., 2016). From the CRCs and PDACs for which we could detect a mutation in the KRAS gene, we were able to confirm its presence in 80% of the CRC-derived (4/5) and 90% of the PDAC-derived (10/11) tumor organoids (Figure 2C). Only a small percentage of PDC models failed to retain the genetic alterations of the original tumors (23% of ccRCC-derived, 20% of the CRC-derived and 10% of the PDAC-derived cell models). In these cases, we often observed overgrowth of benign cells in the cultures thus corroborating previous reports showing that also tissue-derived normal cells can grow efficiently *in vitro* (van de Wetering et al., 2015; Lobo et al., 2016; Drost and Clevers, 2018; Grassi et al., 2019; Saeed et al., 2019). Some PDC models remained, for which single tumor-specific molecular alterations were elusive (6 CRCs, 3 PDACs and 18 RCCs). For these cases, comprehensive molecular profiling remained the only possibility for cell model validation but as this exceeded our possibilities, these samples have not been validated systematically to date. Nevertheless, in our living cell biobank we have successfully established a considerable number of PDC models from several tumor types that closely recapitulate important properties of the original tumors and are therefore valuable tools for translational cancer research and pre-clinical drug profiling (Figure 2C).

## Sample Management is Guided by Policies and Standard Operation Procedures

In order to ensure the availability of these well characterized, annotated and carefully preserved biospecimens for molecular and functional investigations, quality assurance is a key element and requires strict adherence to standard operating procedures (SOPs). Starting at the tissue collection procedure, control of pre-analytic parameters (e.g., relating to the patients, sampling, transport, processing and storage processes) is important because these variables can potentially impact on the outcome of analytical results. Careful annotation of these parameters may be integrated by the Sample PREanalytical Code (SPREC), a comprehensive tool to document pre-analytical details of biospecimens (Lehmann et al., 2012). Another possibility is the retrospective quality stratification that is based on examination and validation of selected samples based on analytical parameters (e.g., RNA integrity) (Betsou et al., 2009). These methods are supported by the reference standard for biobanks ISO 20387 that defines minimum standardization requirements for the organization and processing of biological samples (Coppola et al., 2019). Both SPREC and ISO 20387 are recognized internationally for procedure harmonization for FFPE and fresh frozen tissue samples (Coppola et al., 2019). Importantly, in our biobanking workflow, annotation of several key pre-analytical elements has been implemented. From our clinical information system it is possible to track a number of parameters (e.g., patient data, tissue ischemia times, sampling procedures and sample sizes) while our biobank-specific SOPs clearly define variables concerning tissue processing (e.g., use of specialized reagents, centrifugation speeds and storage conditions) but some other parameters are more difficult to assess (e.g., patient fasting status, transport conditions). However, regulation and recording of pre-analytic variables, even though desirable, has to comply with the circumstances of the clinical workup. Since the impact of many of these parameters is largely unknown to date (Galissier et al., 2016) and native tissue samples for living cell biobanking are less readily available, we most often put emphasize on the possibility of collecting specimens of our interest over the extent of pre-analytical control.

Furthermore, the procurement of patient biospecimens is subject to stringent legal and ethical regulations. Despite the legislative frameworks that apply to biobanking activities varying considerably among the European countries (Kaye et al., 2016), a number of ethical considerations have to be universally addressed. In all legislative systems, the dignity, privacy and health of human beings that donate their tissue have to be protected while at the same time creating a favorable framework for research with human samples (Boers et al., 2016). In Switzerland, the Swiss Human Research Act (HRA) regulates both clinical and non-clinical trials involving human beings and is thus applicable for the collection and utilization of patient tissues and their derivatives for research purposes. For multicenter biobanking projects across Europe, the provisions for the use of tissue samples and data for biomedical research can be found in different sources of law in each jurisdiction (Kaye et al., 2016). In addition, the recently established European General Data Protection Regulation (GDPR) that controls data and privacy protection affects biobanks and their guideline for specimens and personal data usage on the European level. Internationally, collaborative platforms for biobanks (e.g., BBMRI-ERIC-EU, ISBER) will likely play an instrumental role to provide an adequate legal framework for sharing samples and data potentially across many borders. In accordance with the current guidelines in Switzerland, patients at the University Hospital Zürich are asked to consent to the further use of their personal data and tissue samples for biomedical research. From the side of the investigators, ethical approval has to be obtained from the local committee for research projects involving data or samples associated with our biospecimen collection. Moreover, to protect patient privacy, all samples are pseudonymised before researchers can obtain them for analyses (Figure 2D). To ensure correct adherence to these guidelines, specialized personnel like study nurses and biobanking staff are employed and an internal review board that also includes clinicians and scientists assesses all new projects (Figure 2D).

Besides the organizational aspects connected to the ethical implications, documentation of all procedures concerning origin, management and storage of samples is a key factor for successfully biobanking and distribution of well-annotated specimens. At the time when we started to develop our next-generation biobanking strategy, a suitable documentation tool for many aspects of living cell biobanking was not readily available and therefore we developed a dedicated, comprehensive biobanking IT infrastructure (Figure 2D). This infrastructure was designed to interface with the clinical IT management systems and thus allows sample annotation with relevant demographic, pre-analytical and diagnostic information. For the documentation of PDC models, we additionally record growth conditions (2D/3D culture systems, specific media usage), cell morphologies (descriptions and photos) and times of passaging, cryopreservation and defrosting of stored samples. In addition, the storage locations of viably frozen cells and their derivatives (DNA, RNA, protein extracts, etc.) can be tracked and since all records have been de-identified the dedicated biobanking databases for fresh frozen tissue and PDC models can be opened up for external researchers aiming to select a study cohort from the available samples. Notably, in this database also sample distribution is documented thus enabling us to follow-up on data that was generated by external investigators (Figure 2D).

Finally, it is fundamental to our tissue and living cell biobank that specimens and data that we collect and store are effectively utilized to support basic and translational biomedical research. In order to demonstrate such value, our ongoing endeavors are directed toward sustaining and expanding our living cell biobank workflow alongside the traditional tumor tissue biorepository for future cancer research and clinical application.

## Discussion

Advances in molecular medicine have significantly increased our understanding of cancer biology in the last two decades and are spawning a paradigm shift in patient care toward personalized treatment strategies. To realize the promise of precision therapy in the clinics, a strong link between pathological data, molecular alterations, phenotypical consequences and patient outcomes will be instrumental. The comprehensive, tripartite biobanking strategy of collecting FFPE, fresh frozen and patient-derived living cells we have described here offers a toolkit for addressing numerous questions in current and future basic, translational and clinical research. Thus, the extension of traditional patient tumor tissue biobanking with viably frozen PDCs and personalized *in vitro* models amenable for functional assays will likely be a major enabling factor for the discovery and development of new diagnostic tools and precision treatment options.

However, next-generation biobanking adds a new layer of complexity to traditional biospecimen collection as it requires an even higher degree of coordination, resources and expertise. For successful living cell biobanking, culture conditions for PDCs from different tumor types have to be established separately by expert scientific staff and each culture needs to be validated and characterized carefully. It is important to note that growth conditions are major influencing factors during propagation of PDC models as they impact on cellular processes such as replication and cell survival as well as on the genotypic and phenotypic heterogeneity of cell subpopulations. Thus far, only few studies have addressed the representation of tumor subclonal cell populations and their dynamics during PDC culture (Clevers, 2016; Fujii et al., 2016; Li et al., 2018; Bolck et al., 2019). To what extent tumor heterogeneity is changing during prolonged cell culture and how this is influenced by growth conditions will need to be investigated in more detail in order to understand the translational value of these models and to determine best practices. In the meantime, it will be paramount to cryopreserve the original, most heterogeneous populations of tumor and associated cells to ensure that their potential for personalized medicine can be maximized once new technologies emerge. As PDC models are more comprehensively studied, it is likely that more elaborate systems will be generated, for example by co-culturing tissue-specific stroma or immune components or applying microfluidics and organ-on-a-chip technologies (Xu et al., 2014; Horvath et al., 2016). Large collections of viably frozen PDCs like the one described here will provide the necessary tools to enable the development of such assay systems and to explore new frontiers for their use. In this process, it is essential that joint oversight of pathologists and scientists will ensure that the selected biospecimens are representative and adequate for the intended study and all organizational, ethical and legal requirements are met.

## Conclusion

Based on our five years of experiences in next-generation biobanking we illustrate a targeted but scalable workflow that can be adjusted to accommodate a variety of research needs or adapted in other pathology departments. We strongly believe that in conjunction with traditional tissue biobanking, a living cell biobank will constitute an important tool for functional cancer research and personalized medicine which at the same time has the potential to fulfill the current demand for more reliable, patient-specific cancer models. However, it is important to note that for next-generation biobanking, additional considerations and long-term investments (e.g., specialized staff, IT resources, equipment and infrastructure) are required in order to preserve the integrity of these collections for future research. This may necessitate transformational changes in research and infrastructure environments that often go beyond current standards for tissue specimen collection in pathology departments. Nevertheless, next-generation biobanking promises major new opportunities for cancer patients and will most likely play a decisive role in advancing precision medicine.

## Data Availability Statement

The raw data supporting the conclusions of this manuscript will be made available by the authors, without undue reservation, to any qualified researcher.

## Ethics Statement

The studies involving human participants were reviewed and approved by Kantonale Ethikkommission Zürich. The patients/participants provided their written informed consent to participate in this study.

## Author Contributions

HM and PS established the tissue and living cell biobank and provided critical support for all aspects of the biobanking activities. PS and HB devised the main conceptual ideas of this work. SD and PS were responsible for all aspects of collecting fixed and fresh frozen tumor specimens. EG, KM, CP, and HB collected and processed fresh human tumor specimens to generate 2D and 3D patient-derived *in vitro* models. EG, KM, and HB performed all validation experiments and maintained the organizational infrastructure of the living cell biobank. CP reviewed cytopathological and histological slides. HB, SD, and PS established the comprehensive biobanking database. HB wrote the manuscript, while CP, HM, and PS provided conceptual support and assisted in the manuscript writing. All authors discussed the work and commented on the manuscript.

## Conflict of Interest

The authors declare that the research was conducted in the absence of any commercial or financial relationships that could be construed as a potential conflict of interest.
